# BMP7 Activates Brown Adipose Tissue and Reduces Diet-Induced Obesity Only at Subthermoneutrality

**DOI:** 10.1371/journal.pone.0074083

**Published:** 2013-09-16

**Authors:** Mariëtte R. Boon, Sjoerd A. A. van den Berg, Yanan Wang, Jan van den Bossche, Sofia Karkampouna, Matthias Bauwens, Marijke De Saint-Hubert, Geertje van der Horst, Slobodan Vukicevic, Menno P. J. de Winther, Louis M. Havekes, J. Wouter Jukema, Jouke T. Tamsma, Gabri van der Pluijm, Ko Willems van Dijk, Patrick C. N. Rensen

**Affiliations:** 1 Department of Endocrinology and Metabolic Diseases, Leiden University Medical Center, Leiden, The Netherlands; 2 Einthoven Laboratory for Experimental Vascular Medicine, Leiden University Medical Center, Leiden, The Netherlands; 3 Department of Human Genetics, Leiden University Medical Center, Leiden, The Netherlands; 4 Department of Medical Biochemistry, Academic Medical Center, Amsterdam, The Netherlands; 5 Department of Molecular Cell Biology, Leiden University Medical Center, Leiden, The Netherlands; 6 Department of Nuclear Medicine, Maastricht University, Maastricht, The Netherlands; 7 Department of Urology, Leiden University Medical Center, Leiden, The Netherlands; 8 Center for Translational and Clinical Research, University of Zagreb, School of Medicine, Zagreb, Croatia; 9 Department of Cardiology, Leiden University Medical Center, Leiden, The Netherlands; 10 TNO-Biosciences, Leiden, The Netherlands; University of Texas Health Science Center at San Antonio, United States of America

## Abstract

**Background/Aims:**

Brown adipose tissue (BAT) dissipates energy stored in triglycerides as heat via the uncoupling protein UCP-1 and is a promising target to combat hyperlipidemia and obesity. BAT is densely innervated by the sympathetic nervous system, which increases BAT differentiation and activity upon cold exposure. Recently, Bone Morphogenetic Protein 7 (BMP7) was identified as an inducer of BAT differentiation. We aimed to elucidate the role of sympathetic activation in the effect of BMP7 on BAT by treating mice with BMP7 at varying ambient temperature, and assessed the therapeutic potential of BMP7 in combating obesity.

**Methods and Results:**

High-fat diet fed lean C57Bl6/J mice were treated with BMP7 via subcutaneous osmotic minipumps for 4 weeks at 21°C or 28°C, the latter being a thermoneutral temperature in which sympathetic activation of BAT is largely diminished. At 21°C, BMP7 increased BAT weight, increased the expression of *Ucp1*, *Cd36* and hormone-sensitive lipase in BAT, and increased total energy expenditure. BMP7 treatment markedly increased food intake without affecting physical activity. Despite that, BMP7 diminished white adipose tissue (WAT) mass, accompanied by increased expression of genes related to intracellular lipolysis in WAT. All these effects were blunted at 28°C. Additionally, BMP7 resulted in extensive ‘browning’ of WAT, as evidenced by increased expression of BAT markers and the appearance of whole clusters of brown adipocytes via immunohistochemistry, independent of environmental temperature. Treatment of diet-induced obese C57Bl6/J mice with BMP7 led to an improved metabolic phenotype, consisting of a decreased fat mass and liver lipids as well as attenuated dyslipidemia and hyperglycemia.

**Conclusion:**

Together, these data show that BMP7-mediated recruitment and activation of BAT only occurs at subthermoneutral temperature, and is thus likely dependent on sympathetic activation of BAT, and that BMP7 may be a promising tool to combat obesity and associated disorders.

## Introduction

Human adipose tissue is broadly classified as either white adipose tissue (WAT) or brown adipose tissue (BAT). WAT functions as an energy storage depot characterized by a large intracellular lipid droplet per adipocyte and is furthermore a prominent endocrine organ, producing hormones that regulate appetite and satiety [1]. In contrast, BAT is an energy dissipation depot characterized by multi-locular lipid droplets per adipocyte and a wealth of densely packed mitochondria. Uncoupling protein-1 (UCP-1) in these mitochondria uncouples respiration from ATP synthesis, leading to heat production [2].

The most well-known trigger for activation of BAT is cold, which increases sympathetic outflow from the hypothalamic temperature centre towards BAT, leading to release of noradrenalin and increased thermogenesis. Recently, a second alternative pathway was demonstrated to control thermogenesis, in which alternatively activated (M2) macrophages release noradrenalin to activate BAT locally [3].

Fatty acids are an important substrate for BAT thermogenesis. The fatty acids originate from triglyceride (TG)-rich lipoproteins and are released upon local lipoprotein lipase (LPL) activity [4]. Mouse studies have shown that BAT has a great clearance capacity for TG. Cold exposure drastically accelerates plasma TG clearance as a result of increased uptake into BAT and thereby corrects hyperlipidemia in pathophysiological settings [5].

The recent findings that metabolically active BAT stores exist in adult humans and that BAT volume and activity are lower in obese subjects [6]–[8], have increased interest in the therapeutic potential of BAT to combat obesity and related disorders, such as dyslipidemia. In both rodents and humans, brown adipocytes are present in well-localized fat pads, called ‘constitutive BAT’, as well as scattered in other tissues, such as WAT and muscle, there forming a ‘peripheral’ pool of brown adipocytes [9]. The brown adipocytes in WAT are also called ‘brite’ cells because their phenotypic characteristics lie between those of white and brown adipocytes [10]. Though in mice it has been demonstrated that a clear phenotypic distinction exists between adipocytes derived from constitutive BAT depots and brite cells, in humans this is still under debate. One recent study in which human brown fat biopsies were genotyped showed that this brown fat was more resembling the beige fat found in rodent white fat depots rather than the classical brown fat [11]. In contrast, another study showed that although the properties of human brown fat varied substantially between individuals, some human samples did have similarities with rodent constitutive BAT [12]. Thus, although it is clear that human adults possess metabolically active BAT stores, the precise composition as well as the stimuli that induce its formation remain to be determined. Identification of signalling pathways that regulate differentiation of the two types of brown adipocytes could lead to the development of novel therapies for obesity and related disorders.

Bone Morphogenetic Protein 7 (BMP7) is an important inducer of brown adipocyte differentiation. *In vitro* treatment of a variety of precursor cells, such as C3H10T1/2 mesenchymal cells and Myf5+ precursor cells, with BMP7 resulted in the development of fully differentiated brown adipocytes with high UCP-1 expression [13], [14]. Moreover, short-term adenoviral overexpression of BMP7 in different mouse models confirmed the ability of BMP7 to increase interscapular brown fat mass and oxygen consumption [13]. However, the potential of BMP7 to induce BAT activity in a more therapeutic regimen is currently unknown. Moreover, the role of sympathetic activation in the effects of BMP7 on brown fat function and energy metabolism, and the therapeutic potential of BMP7 to treat dyslipidemia and obesity has not been reported yet.

Therefore, the aim of the present study was to elucidate the role of ambient temperature, which determines the sympathetic output to BAT, in the effect of BMP7 on BAT and to asses the therapeutic potential of BMP7 in combating obesity and related disorders. We show here that treatment of C57Bl6/J mice with BMP7 via osmotic minipumps for 4 weeks effectively increased BAT differentiation, BAT activity and total energy expenditure, and decreased white fat mass. These effects were blunted at 28°C (thermoneutral temperature) and are thus likely dependent on the degree of sympathetic activation. Furthermore, BMP7 markedly enhanced brite cell formation in WAT independent of environmental temperature. Of note, treatment of diet-induced obese C57Bl6/J mice with BMP7 diminished fat mass and liver lipid content and attenuated dyslipidemia and hyperglycemia. Together, our results show that low subthermoneutral ambient temperature, at which sympathetic activation is present, is required for BMP7-mediated recruitment and activation of BAT and suggest that BMP7 may be a therapeutic tool to ameliorate obesity, and related disorders.

## Research Design and Methods

### Animals

Male C57Bl/6J mice (Jackson Laboratory, Bar Harbor, ME) were housed in conventional cages with a 12∶12-h light-dark cycle and had free access to food and water. All animal experiments were approved by the institutional ethics committee on animal care and experimentation at Leiden University Medical Center.

#### Mechanistic studies on the effect of BMP7 on BAT

4-week old C57Bl/6J mice were randomized according to their body weight and plasma triglyceride (TG) and total cholesterol (TC) levels into 6 groups (n = 9). Mice were housed at 21°C or at 28°C (i.e. thermoneutral temperature) and received soluble recombinant BMP7 (obtained from the Vukicevic lab, Zagreb, Croatia) (33 µg/kg/day or 100 µg/kg/day) or vehicle (saline) for 4 weeks while being fed a high-fat diet (D12451, Research Diet Services, which contains 4.73 kcal/gram diet and 45% of kcal as fat, 35% as carbohydrate and 20% as protein). BMP7 or saline was administered at a constant rate via an osmotic minipump (model 1004, Alzet DURECT Corp), which was implanted subcutaneously in the left back region. Mice were weighed twice a week and food intake was measured every other day by weighing the food that was left on the racks.

### ^18^F-FDG PET Scans

After 7 days of treatment, mice underwent an ^18^F-fluorodeoxyglucose (FDG) positron emission tomography (PET) scan to quantify the metabolic volume of different BAT depots (interscapular, neck and back). After 2 h fasting, mice were anesthetized using isoflurane and i.p. injected with ^18^F-FDG (20 MBq). Then, mice were allowed to awake, and were placed in their cage for 1 h (phase of tracer uptake). Scans were performed with the microPET system (uPET Focus 120, Siemens). Data for accumulation of ^18^F-FDG on the PET images were determined on the basis of radioactive counts per volume, corrected for the injected dose per gram of animal weight. A volume of interest was manually drawn around the different BAT depots, with a cut-off value of 50% of the maximum inside the volume, to determine BAT metabolic volume.

### Indirect Calorimetry

Indirect calorimetry was performed in fully automatic metabolic cages (LabMaster System, TSE Systems, Bad Homburg, Germany) during the fourth week of treatment. After 20 h acclimatization, oxygen uptake (V˙ O_2_), carbon dioxide production (V˙ CO_2_) and caloric intake were measured for 5 consecutive days. Carbohydrate and fat oxidation rates were calculated from V˙ O2 and V˙ CO2 as described previously [15]. Total energy expenditure (EE) was calculated from the sum of carbohydrate and fat oxidation. Physical activity was measured using infrared sensor frames.

### RNA Isolation and Q-RT-PCR Analysis

Total RNA was isolated with the Nucleospin® RNA II Kit (Macherey-Nagel) according to the manufacturer’s instructions. 1 µg of total RNA was reverse-transcribed with iScript cDNA synthesis kit (Bio-Rad), and the obtained cDNA was purified with Nucleospin Extract II kit (Macherey-Nagel). Real-time PCR was carried out on the IQ5 PCR machine (Bio-Rad) using the Sensimix SYBR Green RT-PCR mix (Quantace). Melt curve analysis was included to assure a single PCR product was formed. Expression levels were normalized using *ß2-microglobulin* and *36b4* as housekeeping genes. Primer sequences are listed in Table 1.

**Table 1:** Primers used for quantitative real-time PCR analysis.

Gene	Forward primer	Reverse primer
*36b4*	GGACCCGAGAAGACCTCCTT	GCACATCACTCAGAATTTCAATGG
*Arg1*	CATGGGCAACCTGTGTCCTT	CGATGTCTTTGGCAGATATGCA
*Atgl*	ACAGTGTCCCCATTCTCAGG	TTGGTTCAGTAGGCCATTCC
*B2-microglobulin*	TGACCGGCTTGTATGCTATC	CAGTGTGAGCCAGGATATAG
*Ccl5*	GGAGTATTTCTACACCAGCAGCAA	GCGGTTCCTTCGAGTGACA
*Cd163*	CTCAGGAAACCAATCCCAGA	CAAGAGCCCTCGTGGTAGAC
*Cd36*	GCAAAGAACAGCAGCAAAATC	CAGTGAAGGCTCAAAGATGG
*Hsl*	AGACACCAGCCAACGGATAC	ATCACCCTCGAAGAAGAGCA
*Il10*	TTTGAATTCCCTGGGTGAGAA	CTCCACTGCCTTGCTCTTATTTTC
*Mrc1*	GAGAGCCAAGCCATGAGAAC	GTCTGCACCCTCCGGTACTA
*Nos2*	CGGGCATCTGGTAGCCAGCG	TGGCAACATCAGGTCGGCCAT
*Soc1*	CCGTGGGTCGCGAGAAC	AAGGAACTCAGGTAGTCACGGAGTA
*Tnf*	GGCAGGTCTACTTTGGAGTCATTGC	ACATTCGAGGCTCCAGTGAATTCGG
*Ucp1*	TCAGGATTGGCCTCTACGAC	TGCATTCTGACCTTCACGAC

*Arg1,* arginase 1; *Atgl,* adipose triglyceride lipase; *Ccl5,* Chemokine (C–C motif) ligand 5;

*Hsl,* hormone-sensitive lipase; *Il10,* interleukin-10; *Mrc1,* mannose receptor 1; *Nos2*,

nitric oxide synthase 2; *Tnf,* tumor necrosis factor-α *Ucp-1,*uncoupling protein-1.

### Histology

Interscapular BAT and gonadal WAT were removed and fixed directly in 4% paraformaldehyde, dehydrated and embedded in paraffin. Sections (5 µm) were dewaxed in xylene, rehydrated in ethanol and treated with 3% H_2_O_2_ (Sigma) in absolute methanol for 30 min. Next, sections were immersed in 10 mM citrate buffer (pH = 6.0), boiled for 10 min and cooled down at room temperature. Slides were blocked during 60 min with normal goat serum (1∶75 in PBS) and incubated overnight at 4°C with rabbit monoclonal anti-UCP-1 antibodies (Abcam) diluted 1∶300 in normal goat serum (1∶75). Next, sections were incubated for 60 min with biotinylated goat α-rabbit secondary antibodies (Vector Labs) diluted 1∶600 in normal goat serum (1∶75). Immunostaining was amplified using Vector Laboratories Elite ABC kit (Vector Labs) and the immunoperoxidase complex was visualized with Nova Red (Vector Labs). Counterstaining was performed with Mayer’s hematoxylin (1∶4).

### Isolation of Peritoneal Macrophages

Mouse peritoneal cells were collected by flushing the peritoneal cavity with 10 ml sterile ice-cold PBS, as previously described [16]. Cells were centrifuged at 1,200 rpm for 5 min at 4°C and resuspended in RPMI1640 medium supplemented with 10% heat-inactivated fecal calf serum. Total leukocyte counts were determined using a Beckman Counter. Cells were plated overnight at 1×10^6^ cells/ml in 500 µl in a 24 wells plate. Cells were subsequently lysed in buffer with β-mercaptoethanol (100∶1) and RNA was isolated as described above.

#### Studies on BMP7 in a diet-induced obesity model

8-week old C57Bl/6J mice were fed a high-fat diet (45% energy, D12451, Research Diet Services) for 12 weeks to induce obesity. Mice were then randomized according to their body weight and plasma TG, TC and glucose levels into groups that received soluble recombinant BMP7 (100 µg/kg/day) or vehicle (saline) for 4 weeks via subcutaneous osmotic minipumps while being fed a high-fat diet. Mice were weighed twice a week and during the fourth week of treatment, mice were house in fully automatic metabolic cages as described under ‘Indirect calorimetry’.

### Dual-energy X-ray Absorptiometry (DEXA) Scan

After 4 weeks treatment, body composition was measured by DEXA using the Norland pDEXA Sabre X-ray Bone Densitometer. Mice were anaesthetized intraperitoneally with a combination of 6.25 mg/kg acepromazine (Alfasan), 6.25 mg/kg midazolam (Roche) and 0.31 mg/kg fentanyl (Janssen-Cilag). The total body of the mice was scanned, yet the heads were excluded from the analyses.

### Plasma Parameters

At baseline and after 4 weeks of treatment, plasma was obtained via tail vein bleeding after 4 h fasting and assayed for TC, TG, and phospholipids using the commercially available enzymatic kits from Roche Molecular Biochemicals. Plasma glucose levels were measured using a commercially available kit and standardized according to the instructions of the manufacturer (Instruchemie, Delfzijl, The Netherlands).

### Liver Lipid Extraction

Lipids were extracted from livers consistent with a modified protocol from Bligh and Dyer [17]. In short, a small piece of liver was homogenized in ice-cold methanol. Lipids were removed after centrifugation by adding 1,800 µl of CH_3_OH-CHCl_3_ (3∶1 vol/vol) to 45 µl of homogenate. The CHCl_3_ phase was dried and suspended in 2% Triton X-100. Hepatic triglyceride (TG), total cholesterol (TC) and phospholipid (PL) concentrations were measured using commercial kits, as explained above. Liver lipids were expressed per milligram of protein, which was determined using the BCA protein assay kit (Pierce).

### *In vitro* Treatment of Bone-marrow Derived Macrophages with BMP7

Bone marrow-derived macrophages were obtained from the hind limbs of C57Bl6/J mice as described previously [16], [18]. All muscle tissue was removed from the bones and the bones were placed in a petri-dish filled with ice-cold sterile PBS, 70% ethanol (30 sec) and then again ice-cold PBS. The femur and tibia were cut at both ends and the bones were flushed through with ice-cold sterile PBS after which the bone marrow was collected. Then, cells were rinsed with PBS, lifted with a warm Lidocain/EDTA/PBS solution, washed in RPMI1640 and plated in LCM at 10^6^ cells/ml in 500 µl in a 24 wells plate for 8 days. At day 9, cells were either stimulated with soluble recombinant BMP7 (8.3 nM) or vehicle for 24 hours or with BMP7 or vehicle for 18 hours +6 hours LPS treatment (10 ng/ml). The supernatant was collected for measurement of IL-6, IL-12 and TNF by commercial ELISA (Invitrogen) and NO_2_^-^ by Griess assay (Invitrogen), according to the manufacturer’s instructions. RNA was isolated with the Roche RNA isolation kit as described by the suppliers.

### Statistical Analysis

All data are represented as means ± SEM unless indicated otherwise. Data were analyzed with SPSS 17.0 using one-way ANOVA (when three groups were compared) Student T-tests (when two groups were compared) or, in case the data were not normally distributed, using nonparametric tests. Statistical analysis for the indirect calorimetry data were performed on 12-hour averages per parameter, based on the light-dark cycle. Data were generated for the light period between 7∶00 AM and 7∶00 PM and for the dark period between 7∶00 PM and 7∶00 AM. Normality checks were performed and comparisons were made using either one-way ANOVA or nonparametric tests. Probability values less than 0.05 were considered statistically significant.

## Results

### BMP7 Increases BAT Volume and UCP-1 Expression at 21°C, but not at Thermoneutrality

**Figure 1:**
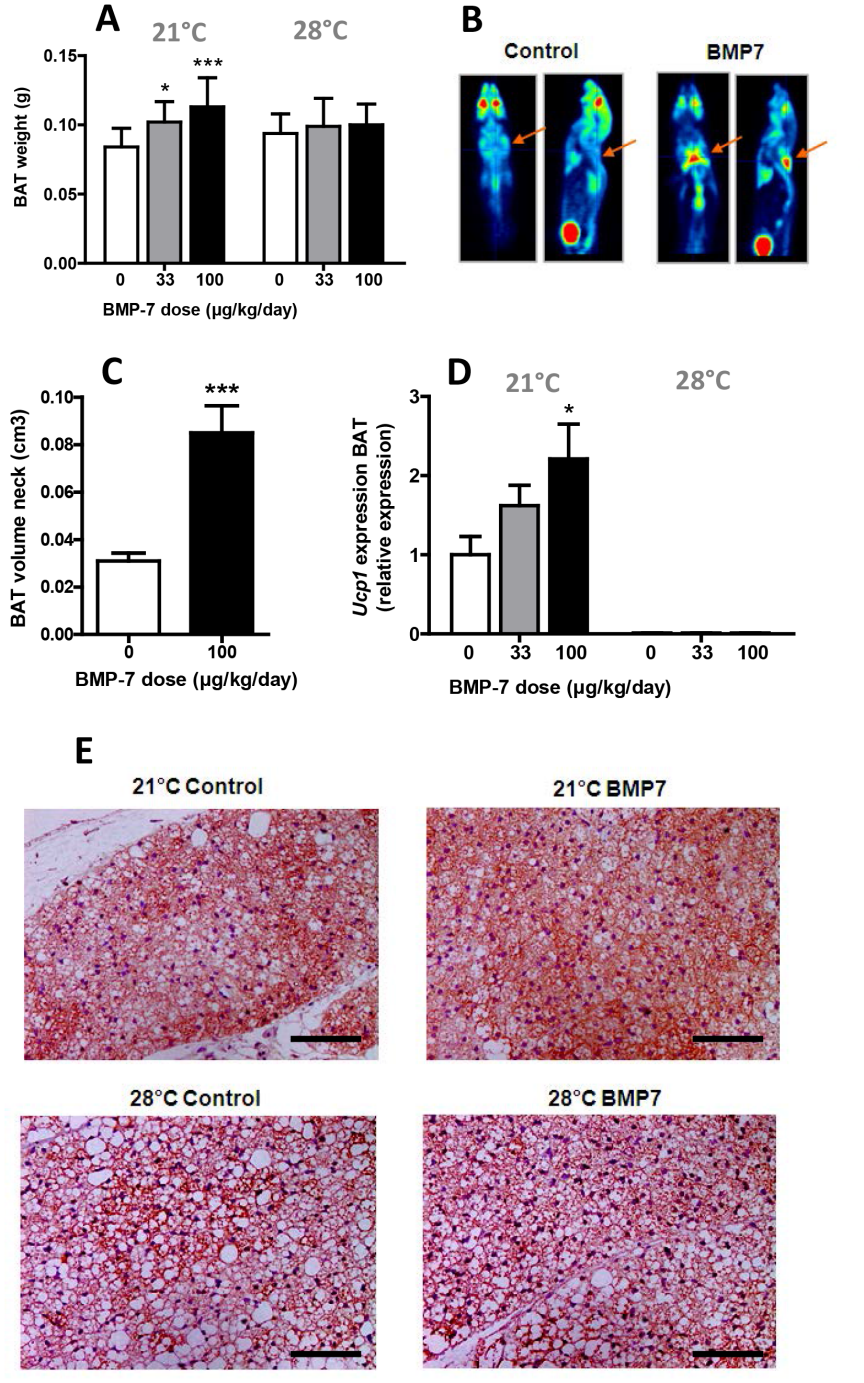
Systemic administration of BMP7 increases BAT volume and UCP-1 expression in mice at 21°C, but not at thermoneutrality. 4-week-old male C57Bl/6J mice were treated for 4 weeks with BMP7 (33 or 100 µg/kg/day) or saline via a subcutaneously located osmotic minipump at an environmental temperature of 21°C or 28°C while feeding a high-fat diet (45% fat). *(A)* Weight of the interscapular brown fat pads, after quantitative removal, of mice housed at 21°C (left) and 28°C (right). *(B)* Representative pictures of ^18^F-FDG PET scans that were taken in control and BMP7 (100 µg/kg/day) treated animals (housed at 21°C) after one week of treatment, from which BAT metabolic volume *(C)* was determined. Arrows indicate neck BAT depots. *(D)* Expression of *Ucp1* in BAT measured by Q-RT-PCR of mice housed at 21°C (left) and 28°C (right). *(E)* Representative pictures of immunohistochemical UCP-1 stainings of BAT in control and BMP7 (100 µg/kg/day) treated animals housed at 21°C (top) and 28°C (bottom). Pink staining represents UCP-1 protein. Magnification 100x. Scale bar, 100 µm. Values are means+SEM (n = 9) and expression of genes was corrected for the housekeeping genes *β2-microglobulin* and *36b4*. *P<0.05, ***P<0.001 compared to the control group.

To investigate whether BMP7 administered via constant low-dose release by osmotic minipumps is capable of increasing BAT volume and activity *in vivo,* 4-week old C57Bl6/J mice (n = 9 per group) were treated with BMP7 at 33 µg/kg/day, 100 µg/kg/day or saline for 4 weeks. Mice were housed at either 21°C or 28°C, the latter being a thermoneutral temperature at which sympathetic activity towards BAT is virtually absent [19]. At 21°C, BMP7 significantly and dose-dependently increased interscapular BAT weight, both at 33 µg/kg/day (+20%, P<0.05) and at 100 µg/kg/day (+30%, P<0.001), while at 28°C no effects of BMP7 were seen (Fig. 1A). ^18^F-FDG PET-scans acquired one week after BMP7 treatment (100 µg/kg/day) in mice that were housed at 21°C, showed that BAT metabolic volume in the neck region had already increased by +175% (P<0.001, Fig. 1B–C). Furthermore, 100 µg/kg/day of BMP7 increased expression of *Ucp1* in BAT as measured by qPCR (+115%, P<0.05, Fig. 1D) and increased UCP-1 protein judged from immunohistochemistry (Fig. 1E). At 28°C, expression of *Ucp1* was markedly downregulated compared to 21°C (−97%, P<0.001), consistent with virtually absent sympathetic activity (Fig. 1D). Moreover, lipid droplet size was markedly increased, pointing to less active BAT (Fig. 1E). Importantly, at 28°C the effect of BMP7 on *Ucp1* expression was completely abolished (Figs. 1D–E).

### BMP7 Dose-dependently Increases Energy Expenditure, Related to Increased Fat Oxidation at 21°C, but not at Thermoneutrality

**Figure 2:**
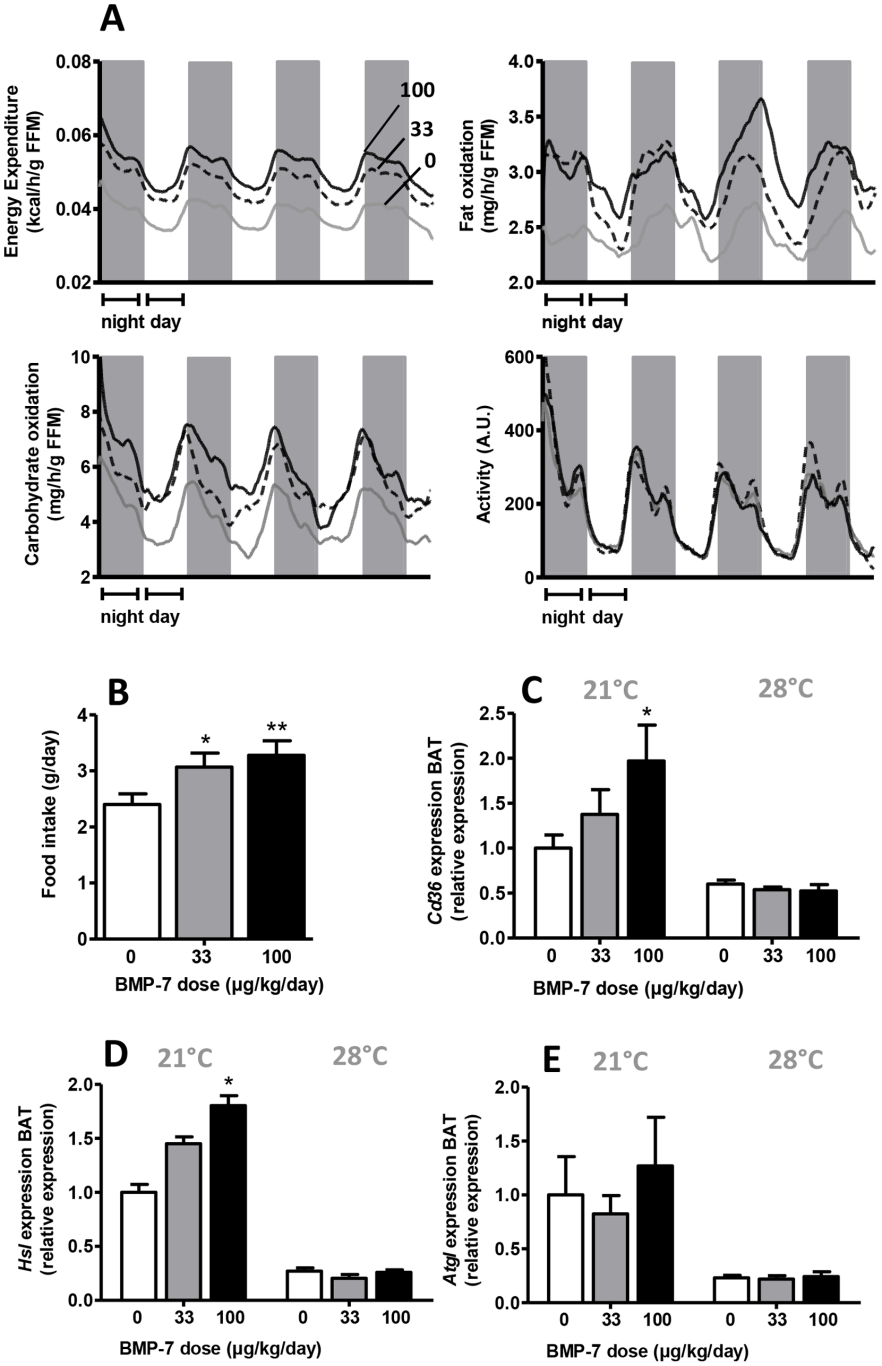
BMP7 increases energy expenditure and fat oxidation at 21°C. 4-week-old male C57Bl/6J mice were treated for 4 weeks with BMP7 (33 or 100 µg/kg/day) or saline via a subcutaneously located osmotic minipump at an environmental temperature of 21°C or 28°C while feeding a high-fat diet (45% fat). *(A)* Energy expenditure, activity levels and fat and carbohydrate oxidation measured during 5 consecutive days in the fourth week of treatment via fully automatic metabolic cages in mice housed at 21°C. Measurements were corrected for free fat mass (FFM) *(B)* Food intake measured during the fourth week of treatment in mice housed at 21°C. *(C–E)* Expression of *Cd36* (*C*), *Hsl* (*D*) and *Atgl* (*E*) in BAT measured by Q-RT-PCR of mice housed at 21°C (left) or 28°C (right). Values are means+SEM (n = 9) and expression of genes was corrected for the housekeeping genes *β2-microglobulin* and *36b4*. *P<0.05, **P<0.01 compared to the control group.

After three weeks of treatment with BMP7, whole body energy metabolism and food intake of mice was assessed with a metabolic cage system. At 21°C, BMP7 significantly and dose-dependently increased energy expenditure, fatty acid and carbohydrate oxidation (up to +45%, +37% and +26% respectively, P<0.05), while physical activity levels were unaltered (Fig. 2A). Because fatty acid and carbohydrate oxidation were both increased, respiratory exchange ratio did not change upon BMP7 treatment (data not shown). Of note, BMP7 treatment resulted in a marked increase in food intake, both at 33 µg/kg/day (+28%, P<0.05) and at 100 µg/kg/day (+37%, P<0.01) (Fig. 2B). At 28°C, total energy expenditure (−24%), fatty acid (−38%) and carbohydrate oxidation (−13%) as well as food intake (−15%) were significantly lower compared to 21°C and the effects of BMP7 were fully absent (Fig. S1A–B). As fatty acids are an important substrate for energy expenditure in BAT, we determined the expression of the scavenger receptor *Cd36*, hormone-sensitive lipase (*Hsl*), and adipose triglyceride lipase (*Atgl*), the latter two involved in intracellular lipolysis. At 21°C, BMP7 increased expression of *Cd36* (up to +95%, P<0.05, Fig. 2C) and *Hsl* (up to +82%, P<0.05, Fig. 2D) but not of *Atgl* (Fig. 2E) in BAT, suggesting increased cellular uptake of fatty acids via CD36 and liberation of intracellular fatty acids from TG via HSL for oxidation purposes. At 28°C, basal expression of *Cd36*, *Hsl* and *Atgl* was lower and unaffected by BMP7 (Figs. 2C–E).

### BMP7 Decreases Gonadal White Fat Weight and Increases Expression of Genes Related to Lipolysis at 21°C, but not at Thermoneutrality

**Figure 3:**
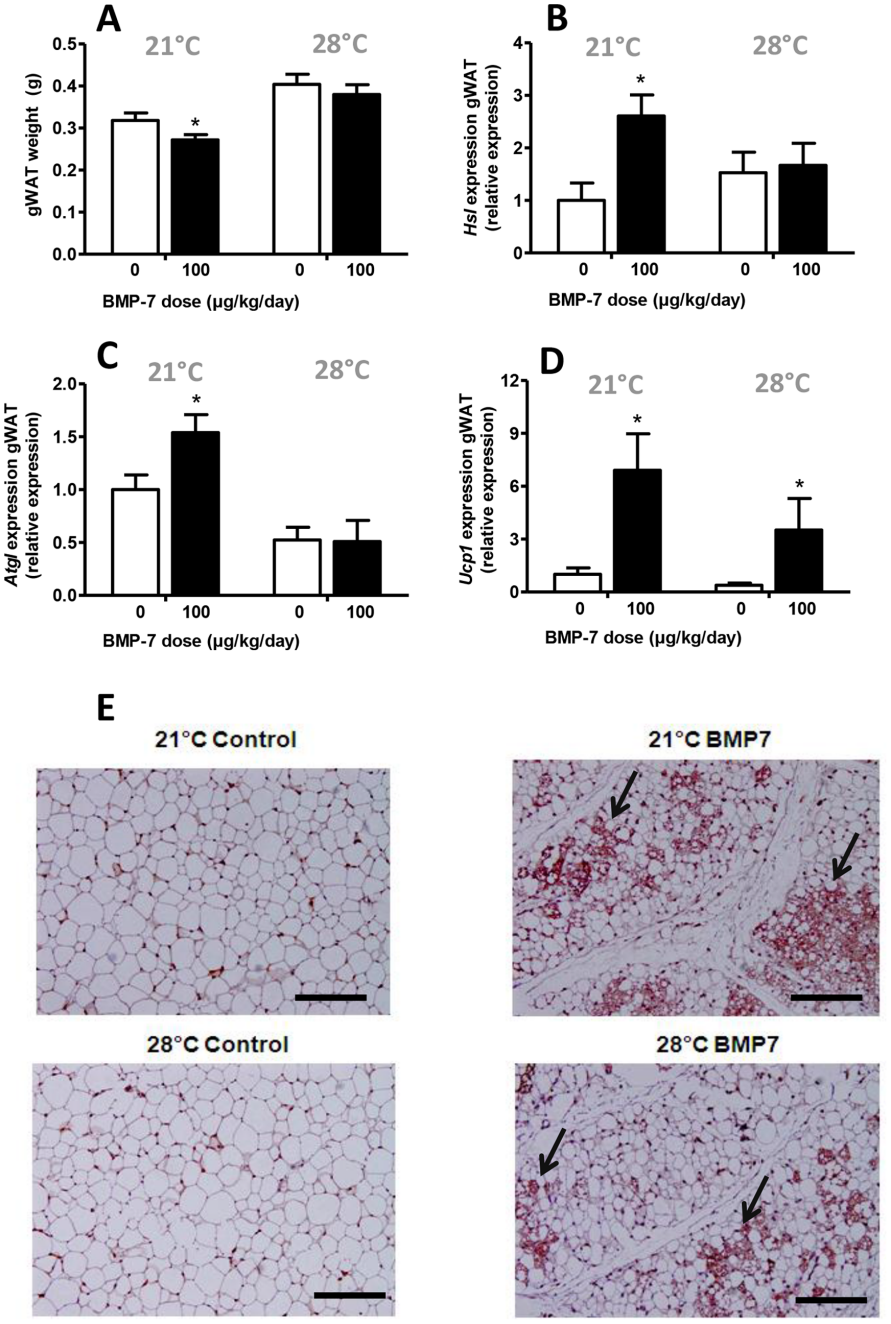
BMP7 reduces white fat weight and induces browning of white adipose tissue. 4-week-old male C57Bl/6J mice were treated for 4 weeks with BMP7 (33 or 100 µg/kg/day) or saline via a subcutaneously located osmotic minipump at an environmental temperature of 21°C or 28°C while feeding a high-fat diet (45% fat). *(A)* Weight of the right gonadal white fat pad, after quantitative removal, after which expression of *Hsl (B), Atgl (C)* and *Ucp1 (D)* was measured by Q-RT-PCR of mice housed at 21°C (left) or 28°C (right). *(E)* Representative pictures of immunohistochemical UCP-1 stainings of WAT in the control and BMP7 (100 µg/kg/day) animals housed at 21°C (top) and 28°C (bottom). Pink staining represents UCP-1 protein. Black arrows point to UCP-1 positive areas. Magnification 100x. Scale bar, 100 µm. Values are means+SEM (n = 9) and expression of genes was corrected for the housekeeping genes *β2-microglobulin* and *36b4*. *P<0.05 compared to the control group.

Despite the increased energy expenditure, BMP7 treatment did not affect body weight development, not at thermoneutrality (Fig. S1C) nor at 21°C (Fig. S2A). This might in part be explained by an increase in bone mineral content induced by BMP7 treatment (+20%, P<0.05) (Fig. S2B), a well-known effect of BMP7 [20]. Of note, mice that were housed at thermoneutrality gained significantly more body weight compared to mice housed at ambient temperature (+25% vs +14%, P<0.05). To investigate whether BMP7 treatment affected white fat content *in vivo*, we quantitatively removed and weighed the right gonadal white fat pad (gWAT). In line with our calorimetric data (Fig. 2A), BMP7 decreased gWAT weight at 21°C (−12%, P<0.05), but not at 28°C (Fig. 3A). To gain more insight into the underlying mechanism, we studied expression of genes related to lipolysis in this fat pad. Indeed, BMP7 increased gene expression of both *Hsl* (+150%, P<0.05, Fig. 3B) and *Atgl* (+50%, P<0.05, Fig. 3C) at 21°C, suggesting increased TG breakdown in WAT, but not at 28°C.

### BMP7 Induces Brite Cell Formation Independent of Environmental Temperature

In WAT, brite cells are present that contribute to total energy expenditure through uncoupling by UCP-1 [21]. Therefore, we studied if BMP7 treatment induced brite cell formation. Indeed, BMP7 markedly increased UCP-1 expression in gWAT, both at 21°C (+920%, P<0.05) and 28°C (+760%, P<0.05) (Fig. 3D). Moreover, immunohistochemical staining of UCP-1 in gWAT confirmed that, at both temperatures, BMP7 induced the appearance of fields of UCP-1 positive cells with brown cell-like morphology, so-called ‘browning’ of white fat (Fig. 3E).

### BMP7 Alters M1/M2 Balance in BAT and WAT at 21°C, but not at Thermoneutrality

**Figure 4:**
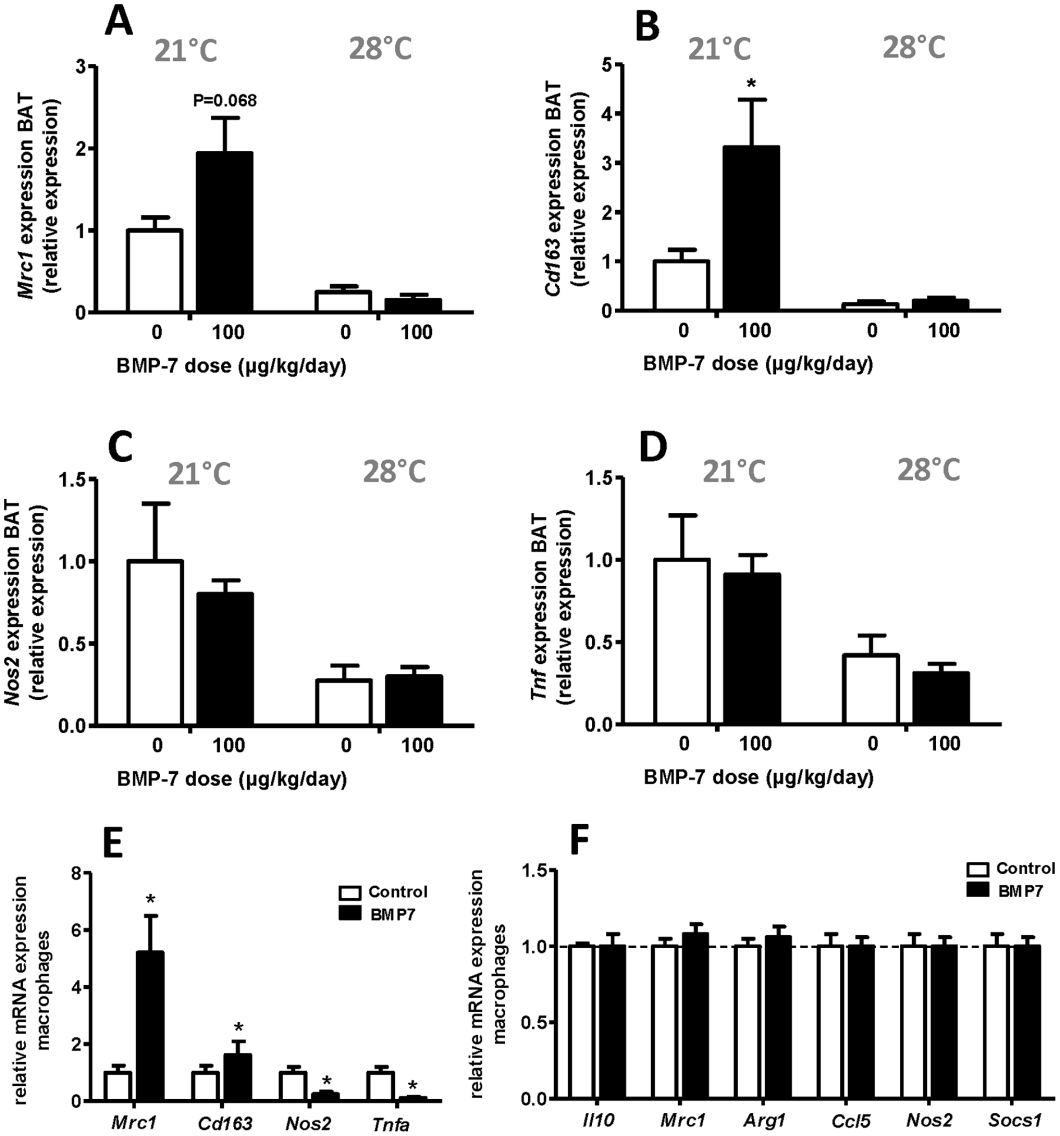
BMP7 results in altered M1/M2 balance in BAT and peritoneal macrophages. 4-week-old male C57Bl/6J mice were treated for 4 weeks with BMP7 (33 or 100 µg/kg/day) or saline via a subcutaneously located osmotic minipump at an environmental temperature of 21°C or 28°C while feeding a high-fat diet (45% fat). Expression of the M2 markers *Mrc1 (A)* and *Cd163 (B)* and the M1 markers *Nos2 (C)* and *Tnf (D)* was measured in BAT by Q-RT-PCR of mice housed at 21°C (left) and 28°C (right). *(E)* After 4 weeks of treatment, peritoneal macrophages were isolated and markers of M2 and M1 macrophages were measured by Q-RT-PCR of mice housed at 21°C. *(F)* Bone-marrow derived macrophages were isolated from untreated C57Bl/6J mice and treated *ex vivo* with BMP7 (8.3 nM) for 24 hrs. The expression of markers for M2 and M1 macrophages was measured via Q-RT-PCR. Values are means+SEM (n = 9 for *in vivo* and n = 3 for *in vitro*) and expression of genes was corrected for the housekeeping genes *β2-microglobulin* and *36b4*. *P<0.05 compared to the control group.

Recently, M2 macrophages were shown to be crucial for BAT function via release of noradrenalin [3]. Recruitment and activation of these macrophages is induced by sympathetic stimulation of BAT [3]. Therefore, we investigated whether BMP7 could influence the presence of M2 macrophages in BAT, thereby possibly contributing to BMP7-induced BAT activation. Indeed, after 4 weeks of BMP7 treatment, expression of the M2 markers *Mrc1* tended to and *Cd163* was increased in BAT (+94%, P = 0.068 and +232%, P<0.05 respectively) (Fig. 4A–B), while the M1 markers *Nos2* and *Tnf* (Fig. 4C–D) were unaltered. Interestingly, at 28°C, basal expression of all tested macrophage markers was largely diminished, which is in line with diminished sympathetic activation, and expression was not influenced by BMP7 treatment (Fig. 4B,D). Moreover, mice that received BMP7 treatment showed increased expression of M2 markers in gWAT, but only at 21°C (Figs. S3A–C). In addition, isolated peritoneal macrophages from mice that had been treated with BMP7 (100 µg/kg/day) at 21°C showed increased expression of the M2 markers *Mrc1* and *Cd163*, while expression of the M1 markers *Tnf* and *Nos2* was markedly diminished (Fig. 4E). Thus, both in the adipose tissue and in the peritoneal cavity, the M1/M2 balance was altered towards M2 after *in vivo* BMP7 treatment at 21°C.

To investigate whether BMP7 directly affects macrophage polarisation, we treated bone-marrow derived macrophages with BMP7 for 24 h *in vitro*. However, no effect was seen on expression of M1 or M2 markers as measured via Q-RT-PCR (Fig. 4F), nor on cytokine production by macrophages after stimulation with LPS (Figs. S4A–D), suggesting that BMP7 alters M1/M2 balance *in vivo* via an indirect mechanism.

### BMP7 Decreases Obesity and Attenuates Liver Lipid Accumulation, Dyslipidemia, and Hyperglycemia in Diet-induced Obese Mice

**Figure 5:**
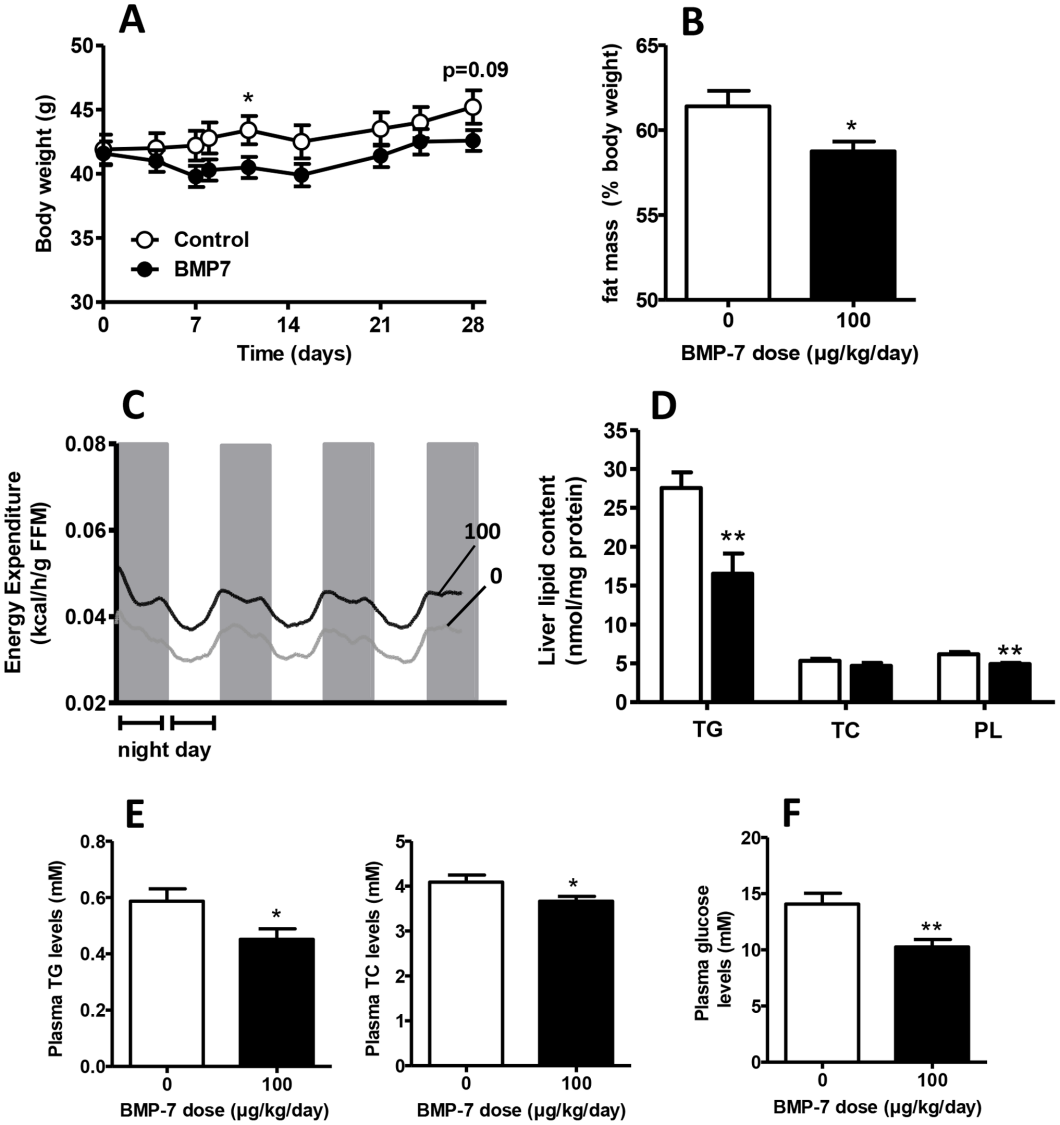
BMP7 decreases obesity and attenuates liver lipid accumulation, dyslipidemia and hyperglycemia in diet-induced obese mice. 8-week-old male C57Bl/6J mice were fed a high-fat diet (45% fat) for 12 weeks and then treated for 4 weeks with BMP7 (100 µg/kg/day) or saline via a subcutaneous osmotic minipump at an environmental temperature of 21°C while feeding a high-fat diet *(A)* Body weight development (gram) *(B)* Total fat mass as measured via DEXA-scan (expressed as percentage of body weight) *(C)* Energy expenditure measured during 5 consecutive days in the fourth week of treatment via fully automatic metabolic cages *(D)* Liver content of triglycerides (TG), total cholesterol (TC) and phospholipids (PL) *(E)* Plasma triglyceride (TG) and total cholesterol (TC) levels. *(F)* Plasma glucose levels. Values are means+SEM (n = 9). *P<0.05, **P<0.01 compared to the control group.

To investigate if BMP7 could improve the metabolic phenotype in diet-induced obesity, C57Bl6/J mice were fed a high-fat diet for 12 weeks and then treated with BMP7 (100 µg/kg/day) or saline for four weeks via subcutaneous osmotic minipumps in the presence of a high-fat diet. After 4 weeks of treatment, BMP7 tended to decrease body weight (−7%, P = 0.09) (Fig. 5A) but significantly reduced total fat mass (−7%, P<0.05) as measured by DEXA analysis (Fig. 5B). Furthermore, BMP7 increased total energy expenditure (+28%, P<0.05) (Fig. 5C). In liver, BMP7 markedly reduced triglyceride (TG) accumulation (−40%, P<0.01) as well as phospholipids (−21%, P<0.05), but not total cholesterol (TC) (Fig. 5D). In addition, BMP7 lowered plasma TG (−25%, P<0.05) and total cholesterol levels (−10%, P<0.05) (Fig. 5E), and diminished hyperglycemia (−27%, P<0.01). Overall, these data are in full accordance with increased fatty acid and carbohydrate oxidation induced by BMP7 treatment and underscore the therapeutic potential of BMP7 to diminish diet-induced obesity and related disorders.

## Discussion

In this study, we show that 4 weeks treatment of mice with the endogenous growth factor BMP7 effectively increased BAT volume and formation of brite cells in WAT and boosted whole-body metabolism. We furthermore demonstrate that at thermoneutral temperature nearly all effects of BMP7 on BAT were absent, suggesting that sympathetic activation at least in part contributes to the effect of BMP7 on BAT differentiation and activity. Moreover, BMP7 was able to diminish white fat content, liver lipid accumulation as well as to reduce dyslipidemia and hyperglycemia in diet-induced obese mice, underscoring the therapeutic potential of BMP7 in combating obesity and related disorders.

Our data are in line with a previous study performed by Tseng et al. [13], in which transient adenoviral overexpression of BMP7 increased BAT volume and oxygen consumption. We show that BMP7 markedly stimulates BAT volume and activity in mice only at subthermoneutral temperature, accompanied by a reduction in diet-induced obesity, liver lipid accumulation, dyslipidemia and hyperglycemia, and an altering of the M1/M2 balance in BAT and WAT towards increased anti-inflammatory M2 macrophages.

Interestingly, we found that treating mice for 4 weeks with BMP7 not only diminished white fat content in diet-induced obese mice, but also markedly lowered liver lipid accumulation, plasma TG levels and, to a lesser extent, plasma cholesterol levels. The lowering in plasma lipid levels is probably the direct consequence of the increased BAT activity induced by BMP7. That is, fatty acids derived from LPL-mediated hydrolysis of TG-rich lipoproteins form an important substrate for BAT thermogenesis [2]. Indeed, expression of CD36 in BAT, a scavenger receptor that is rate-limiting for the uptake of fatty acids from plasma into BAT [22], was significantly upregulated in BAT of BMP7 treated mice. Moreover, a recent study by Bartelt et al. [5] shows that BAT has a tremendous capacity to clear plasma TG. 4 h of cold induction (4°C) normalized plasma TG levels in severely hypertriglyceridemic apoA5 knock-out mice [5]. Since increased plasma TG levels are an independent risk factor for cardiovascular disease in both men and women [23] BMP7 might thus be an interesting treatment modality to manage cardiovascular disease through attenuating dyslipidemia via targeting BAT.

Despite an improved metabolic phenotype in both lean and diet-induced obese mice upon BMP7 treatment, we did not observe effects on body weight development. This might in part be due to the fact that BMP7 is capable of inducing bone formation [20]. Indeed, in our study, BMP7 treated mice exhibited increased bone mineral content as measured via DEXA-scan. In addition, part of the beneficial effect of BMP7 on energy expenditure might be offset by the increase in food intake (up to +37%) we observed. This is probably rather a consequence of the increased uncoupling due to BAT activation [24] then a direct effect of BMP7 on food intake, since we did not observe this effect at thermoneutrality. Moreover, a recent study by Townsend et al [25] showed that intracerebral infusion of BMP7 resulted in a decrease in food intake rather than an increase.

A striking finding in our study is that at thermoneutrality, BMP7 exerted virtually no effect on BAT, suggesting that sympathetic activation of BAT is a prerequisite for the effects of BMP7 on BAT differentiation and activity.

A first explanatory mechanism for this finding may involve the intracellular signaling route by which BMP7 induces UCP-1 upregulation in brown adipocytes. The pathway involves binding of BMP7 to the BMP-receptor II subtype and induction of the p38 MAPK route. This results, via activation of still unknown transcription factors, in upregulation of UCP-1 expression [26], [27]. Interestingly, the β3-adrenergic signaling route, which is induced upon sympathetic activation of BAT, also induces the p38 MAPK route, though via protein kinase A (PKA) activation [27]. Thus, via p38 MAPK, the β3-adrenergic and BMP7 signaling routes are intertwined. Possibly, a certain degree of p38 MAPK activity is essential for the BMP7 signaling route to induce its downstream effects on UCP-1 expression. This might explain why at thermoneutrality, in which sympathetic activity and thus β3 signalling towards BAT are largely diminished, BMP7 was unable to increase BAT volume and activity.

A second mechanism that could explain the ineffectiveness of BMP7 at thermoneutral temperature may involve a central mode of action of BMP7. A central player involved in BAT activation is the hypothalamus, which projects onto sympathetic nerves that densely innervate BAT [2]. Various circulating peptides have been shown to be capable of activating BAT via the hypothalamus, such as GLP-1 [28] and BMP8B, another member of the BMP family [29]. In addition, Townsend et al. [25] showed that central administration of BMP7 resulted in reduced food intake, confirming that BMP7 is at least capable of exerting central effects. However, it remains to be determined whether subcutaneously administered BMP7 is able to enter the hypothalamus to subsequently exert central actions. The absence of effect of BMP7 at thermoneutral temperature can then be explained by largely reduced sympathetic output towards BAT.

Interestingly, we showed that treatment of mice with BMP7 resulted in an altered M1/M2 macrophage balance in both BAT, gWAT and the peritoneal cavity, with increased expression of M2 markers. M2 macrophages have recently been shown to importantly contribute to BAT thermogenesis by releasing noradrenalin [3]. Possibly the increased presence of M2 macrophages in BAT in response to BMP7 treatment contributed to the increased BAT activity found in these mice. However, since the initial paper describing the involvement of M2 macrophages in BAT function has been under debate recently, future studies should further explore the necessity of M2 macrophages in mediating the effects of BMP7 on BAT. Furthermore, whether the effect of BMP7 on macrophage polarisation is either direct or indirect, in response to changes in BAT, remains to be determined. Although we could not show in our *in vitro* experiments a direct effect of BMP7 on M1/M2 skewing in bone-marrow derived macrophages, a recent study in which human THP-1 monocytes were treated with BMP7 demonstrated significant polarization of monocytes into M2 macrophages [30]. However, since the change in M1/M2 balance did not happen at thermoneutral temperature suggests that environmental factors are at least in part involved in the effects of BMP7 on macrophage polarisation.

In this study, we show that BMP7 not only increased the volume of the interscapular brown fat pad, but also show for the first time that BMP7 markedly induced appearance of brite cells in WAT, so-called ‘browning’ of WAT. This could have been caused by either transdifferentiation of white fat cells into brite adipocytes, or differentiation of brite precursor cells that are present in WAT towards brite adipocytes. Both mechanisms are plausible. White fat cells have been shown to be capable of transdifferentiating towards brite adipocytes and they furthermore have the BMP-II receptor [21], [31]. Moreover, in WAT, Myf5- precursor cells are present that can differentiate into brite cells *in vitro* [9], [32]. Future studies are needed to elucidate the mechanism by which BMP7 induces ‘browning’ of WAT in more detail. However, since we found that brown fat cells obviously appeared as clusters in WAT, it is more plausible that BMP7 primarily acted on precursor cells resulting in proliferation and subsequent differentiation. Intriguingly, browning happened independent of environmental temperature. This could be explained by the fact that WAT, in contrast to BAT, is less dense innervated by the sympathetic nervous system and thus probably less dependent on its activation [2]. In addition, UCP-1 expression might be differentially regulated in bright cells compared to the brown adipocytes present in the brown fat pads.

Brite cells have been suggested to contribute importantly to total energy expenditure, as these cells are more abundant in obesity-resistant strains of mice [33], [34]. In our study, mice that were treated with BMP7 at 28°C displayed an increase of brite fat cells without activating brown fat pads. This was however not sufficient to raise total energy expenditure. Interestingly, the recently indentified hormone irisin, which is released from muscle after exercise, selectively induced browning of subcutaneous WAT depots, without affecting differentiation or activity of brown fat pads, and did lead to an increase in total energy expenditure [20].

In conclusion, BMP7 stimulates BAT volume, activity and total energy expenditure only at subthermoneutrality, suggesting that intact sympathetic activation is a prerequisite for the effects of BMP7 on BAT. Furthermore, we found that BMP7 diminishes obesity and liver lipid accumulation, and attenuates dyslipidemia and hyperglycemia in diet-induced obese mice. We anticipate that BAT may be a promising novel treatment goal, and BMP7 a treatment modality, in fighting obesity and related disorders.

## Supporting Information

Figure S1 BMP7 does not affect energy expenditure, fat oxidation, food intake and weight development at thermoneutrality. 4-week-old male C57Bl/6J mice were treated for 4 weeks with BMP7 (33 or 100 µg/kg/day) or saline via a subcutaneously located osmotic minipumps at an environmental temperature of 21°C or 28°C while feeding a high-fat diet (45% fat). *(A)* Energy expenditure, fat and carbohydrate oxidation and activity levels measured during 5 consecutive days in the fourth week of treatment via fully automatic metabolic cages in mice housed at 28°C. Measurements were corrected for free fat mass (FFM). *(B)* Food intake measured during the fourth week of treatment in mice housed at 28°C. *(C)* Body weight (gram) development during treatment at 28°C. Values are means+SEM (n = 9).
(TIF)

Figure S2 BMP7 does not affect weight development but increases bone mineral content at 21°C. 4-week-old male C57Bl/6J mice were treated for 4 weeks with BMP7 (33 or 100 µg/kg/day) or saline via a subcutaneously located osmotic minipumps at an environmental temperature of 21°C or 28°C while feeding a high-fat diet (45% energy). *(A)* Body weight development (gram) during treatment at 21°C *(B)* Bone mineral content as measured via DEXA scan after 4 weeks of treatment at 21°C. Values are means+SEM (n = 9) *P<0.05 compared to the control group.
(TIF)

Figure S3 BMP7 alters the M1/M2 balance in WAT at 21°C, but not at thermoneutrality. 4-week-old male C57Bl/6J mice were treated for 4 weeks with BMP7 (33 or 100 µg/kg/day) or saline via a subcutaneously located osmotic minipump at an environmental temperature of 21°C or 28°C while feeding a high-fat diet (45% energy). *(A–B)* Expression of the M2 markers *Mrc1 (A)* and *Cd163* (*B)* in gWAT measured by Q-RT-PCR of mice housed at 21°C (left) or 28°C (right). *(C)* Expression of the M1 marker *Nos2* in gWAT measured by Q-RT-PCR of mice housed at 21°C (left) or 28°C (right). Values are means+SEM (n = 9) and expression of genes was corrected for the housekeeping genes *β2-microglobulin* and *36b4*. *P<0.05 compared to the control group.
(TIF)

Figure S4 BMP7 does not alter cytokine secretion by bone-marrow derived macrophages. Bone-marrow derived macrophages were isolated from untreated male C57BL6/J mice, plated in a 24-well plate (10^6^ cells/mL) and stimulated with BMP7 (8.3 nM) or vehicle for 24 hours or with BMP7 (8.3 nM) or vehicle for 18 hours +6 hours LPS (10 ng/mL). The concentration of IL-6 *(A)*, TNF *(B),* NO_2_^-^ *(C)* and IL-12 *(D)* in the supernatant was measured. Values are means+SEM (n = 3).
(TIF)
