# Supplementary figures and images for: BMP7 Activates Brown Adipose Tissue and Reduces Diet-Induced Obesity Only at Subthermoneutrality
Source: PLoS One. 2013 Sep 16;8(9):e74083. doi: 10.1371/journal.pone.0074083 (PMC3774620; doi:10.1371/journal.pone.0074083)

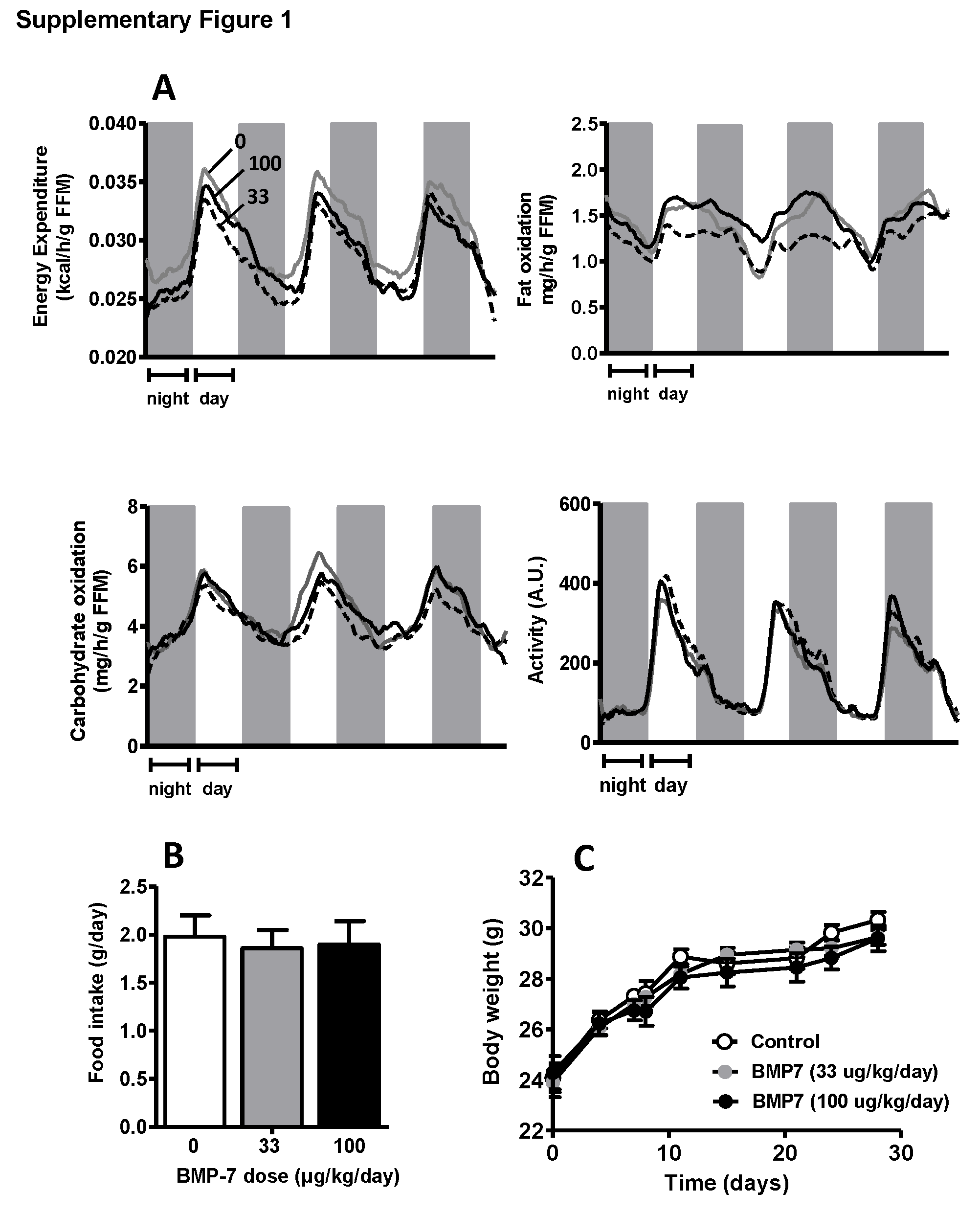

Supplement: Figure S1 — BMP7 does not affect energy expenditure, fat oxidation, food intake and weight development at thermoneutrality. 4-week-old male C57Bl/6J mice were treated for 4 weeks with BMP7 (33 or 100 µg/kg/day) or saline via a subcutaneously located osmotic minipumps at an environmental temperature of 21°C or 28°C while feeding a high-fat diet (45% fat). (A) Energy expenditure, fat and carbohydrate oxidation and activity levels measured during 5 consecutive days in the fourth week of treatment via fully automatic metabolic cages in mice housed at 28°C. Measurements were corrected for free fat mass (FFM). (B) Food intake measured during the fourth week of treatment in mice housed at 28°C. (C) Body weight (gram) development during treatment at 28°C. Values are means+SEM (n = 9). (TIF) [file pone.0074083.s001.tif]

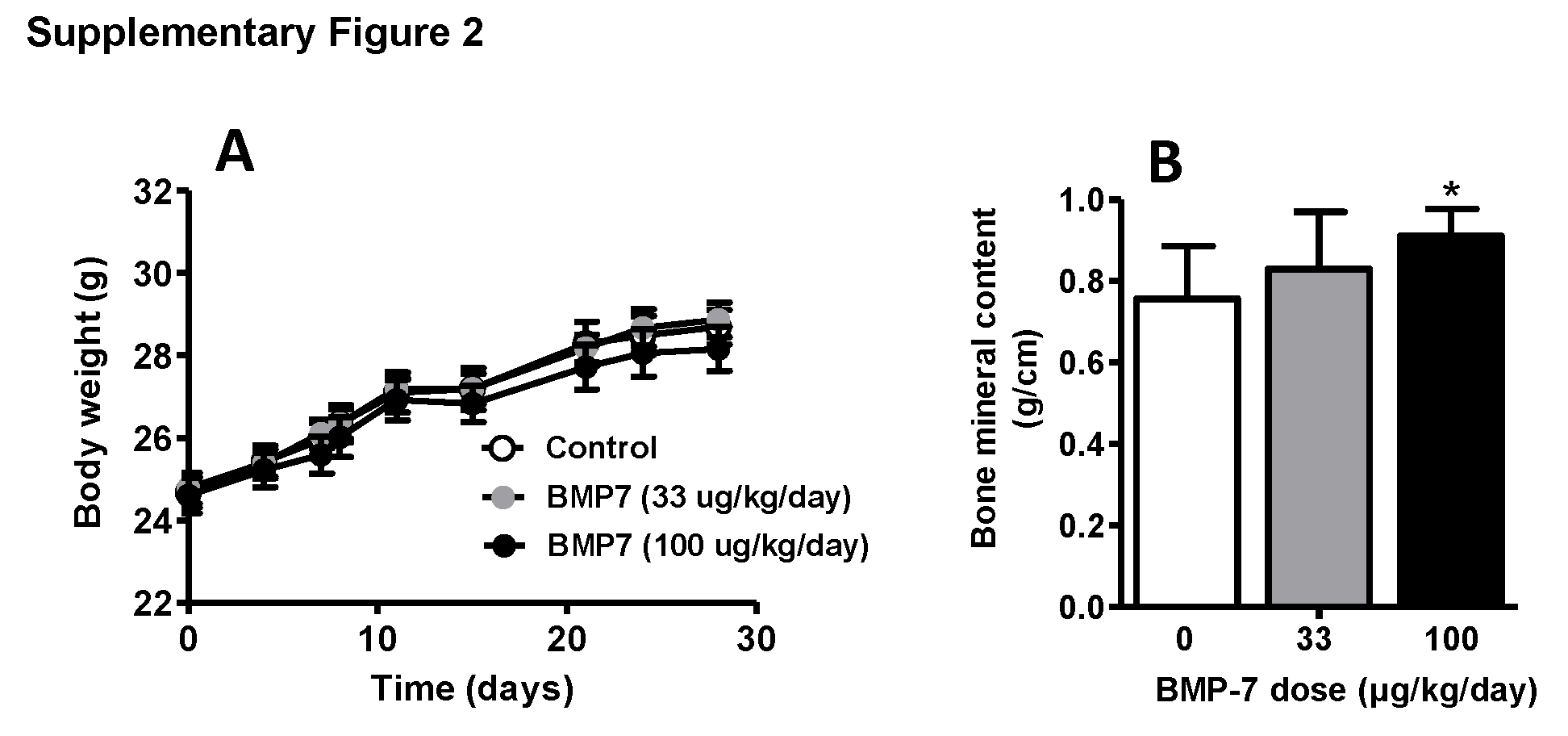

Supplement: Figure S2 — BMP7 does not affect weight development but increases bone mineral content at 21°C. 4-week-old male C57Bl/6J mice were treated for 4 weeks with BMP7 (33 or 100 µg/kg/day) or saline via a subcutaneously located osmotic minipumps at an environmental temperature of 21°C or 28°C while feeding a high-fat diet (45% energy). (A) Body weight development (gram) during treatment at 21°C (B) Bone mineral content as measured via DEXA scan after 4 weeks of treatment at 21°C. Values are means+SEM (n = 9) *P<0.05 compared to the control group. (TIF) [file pone.0074083.s002.tif]

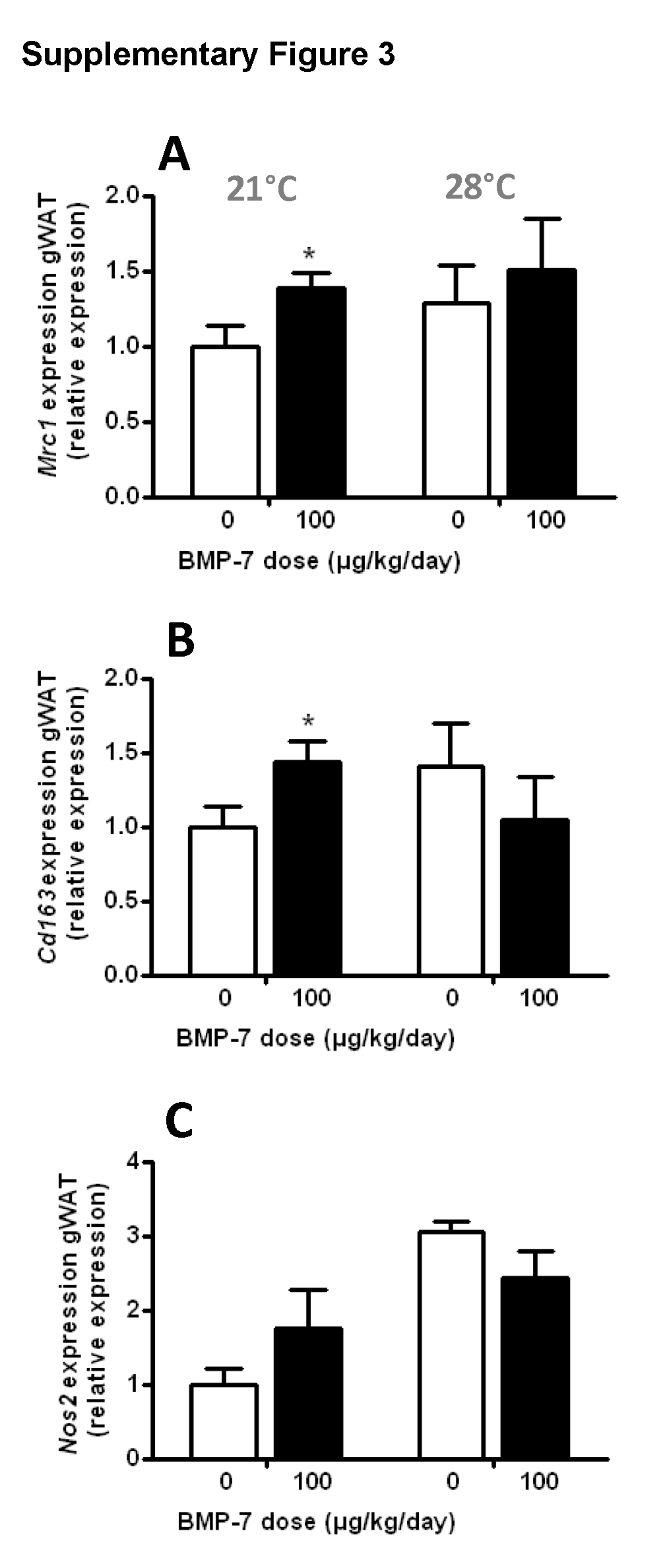

Supplement: Figure S3 — BMP7 alters the M1/M2 balance in WAT at 21°C, but not at thermoneutrality. 4-week-old male C57Bl/6J mice were treated for 4 weeks with BMP7 (33 or 100 µg/kg/day) or saline via a subcutaneously located osmotic minipump at an environmental temperature of 21°C or 28°C while feeding a high-fat diet (45% energy). (A–B) Expression of the M2 markers Mrc1 (A) and Cd163 (B) in gWAT measured by Q-RT-PCR of mice housed at 21°C (left) or 28°C (right). (C) Expression of the M1 marker Nos2 in gWAT measured by Q-RT-PCR of mice housed at 21°C (left) or 28°C (right). Values are means+SEM (n = 9) and expression of genes was corrected for the housekeeping genes β2-microglobulin and 36b4. *P<0.05 compared to the control group. (TIF) [file pone.0074083.s003.tif]

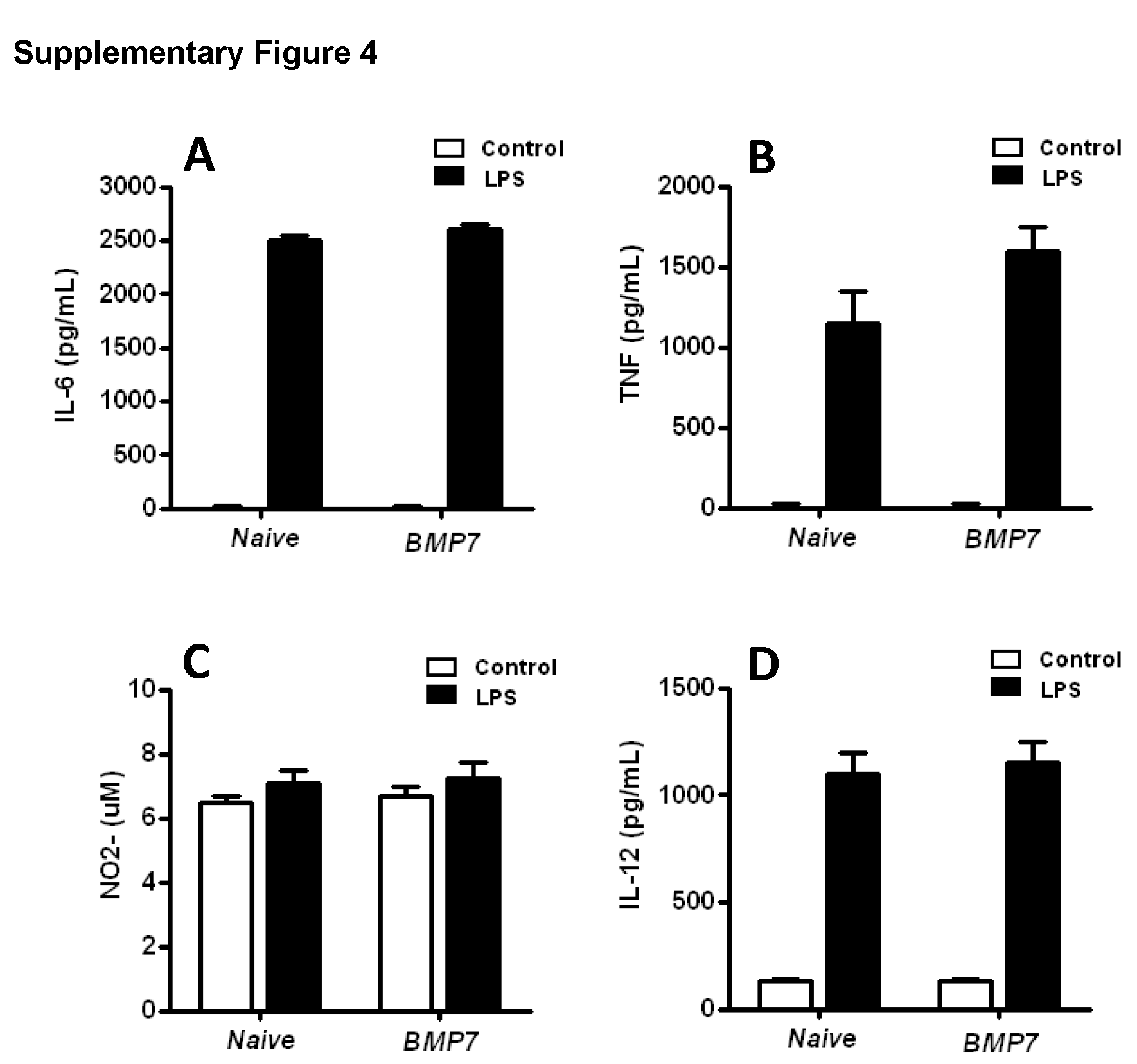

Supplement: Figure S4 — BMP7 does not alter cytokine secretion by bone-marrow derived macrophages. Bone-marrow derived macrophages were isolated from untreated male C57BL6/J mice, plated in a 24-well plate (106 cells/mL) and stimulated with BMP7 (8.3 nM) or vehicle for 24 hours or with BMP7 (8.3 nM) or vehicle for 18 hours +6 hours LPS (10 ng/mL). The concentration of IL-6 (A), TNF (B), NO2 - (C) and IL-12 (D) in the supernatant was measured. Values are means+SEM (n = 3). (TIF) [file pone.0074083.s004.tif]
